# Histopathological Characteristics and Multi-Omics Analysis of Ocular Pigmentation Defects in Albino *Percocypris pingi*

**DOI:** 10.3390/cells14171377

**Published:** 2025-09-04

**Authors:** Senyue Liu, Xiaoyun Wu, Qiaolin Zou, Jiansheng Lai, Yongqiang Deng, Yang Feng, Chengyan Mou, Mingjiang Song, Pengcheng Li, Jun Du, Yan Liu, Qiang Li, Ya Liu

**Affiliations:** 1 Fisheries Research Institute, Sichuan Academy of Agricultural Sciences (Sichuan Fisheries Research Institute), Chengdu 611731, China; liusenyue@scsaas.cn (S.L.);; 2 Aquatic Health and Intelligent Aquaculture Key Laboratory of Sichuan Province, Chengdu 611130, China

**Keywords:** albinism, pigment, ocular albinism, multiomics, *Percocypris pingi*

## Abstract

*Percocypris pingi* was listed in the China Vertebrate Red List in 2015, and albino *P. pingi* exhibits remarkable ocular phenotypes due to melanin synthesis defects, including the deficiency of melanin granules in the iris and retinal pigment epithelium (RPE). However, the regulatory mechanism of pigment loss in the eyes of albino *P. pingi* has not yet been clarified. This study systematically revealed the potential mechanisms underlying the obstruction of ocular melanin synthesis in albino *P. pingi* through histopathological analysis, transcriptomics, and proteomics techniques. The results showed that the synergistic effects of abnormal H^+^ transport mediated by SLC45A2, excessive activation of retinol metabolism, and cytoskeletal transport disorders led to the inhibition of tyrosinase activity and retention of pigment granules, ultimately causing melanin deficiency in the eyes. This study first elucidates the molecular network of ocular albinism in fish from a multi-omics perspective, providing a new perspective for the mechanistic research of pigmentation disorders in vertebrates.

## 1. Introduction

Melanin, a pigment granule synthesized in melanocytes [1], is primarily categorized into two types: black/brown eumelanin and yellow/red pheomelanin [2]. As one of the most critical pigments in vertebrates, the precise regulation of melanin synthesis, transportation, and deposition not only determines the body color phenotypes of species but also is intimately associated with physiological functions such as visual system development, immune response, and environmental adaptability [3]. Specifically, melanin is synthesized within melanosomes of melanocytes via enzymatic reactions such as tyrosinase catalysis. After generation, it is transported to the stratum corneum or retinal pigment epithelium (RPE), thereby achieving pigmentation [4].

Oculocutaneous albinism (OCA) is a rare disorder caused by impaired melanin synthesis, leading to hypopigmentation in the skin and eyes. Its core mechanisms are closely associated with the disorder of the tyrosine metabolic pathway, the defect of pigment granule transport, and abnormal expression of related genes [5]. However, existing studies are primarily focused on the albino mechanisms of mammalian skin or hair (such as loss of tyrosinase activity caused by tyrosine (TYR) and OCA2 gene mutations) [6], leaving significant research gaps in the analysis of the molecular regulatory network of albinism in fish, especially ocular albinism. As independent pigment regulation units, the pigment regulation mechanisms of the ocular microenvironment (such as pH homeostasis and retinol metabolic cycle) may differ significantly from those of skin tissues [5]. Notably, fish have been confirmed to possess six types of pigmentation-regulating cells (melanophores, xanthophores, iridophores, leucophores, erythrophores, and cyanophores) [7], suggesting that the regulatory mechanisms of pigmentation in fish may be more complex than those in mammals.

*Percocypris pingi* (*P. pingi*) was listed in the China Vertebrate Red List in 2015 [8]. As an endangered fish species under key protection in China, the conservation and genetic improvement of its wild population are of urgent scientific significance [9]. The wild-type *P. pingi*, characterized by dark brown spots on the body surface and deep pigment deposition in the iris, serves as an ideal model for studying vertebrate pigment metabolism. Apart from the wild type, the appearance of the albino mutant also holds significant research value, as it provides crucial materials for genetic breeding studies. The ocular pigment synthesis defects in albino mutants (such as iris transparency, lack of pigment granules in the RPE, and red-eye phenotype) are highly similar to human ocular albinism [10], but its molecular mechanisms remain unelucidated.

Therefore, in this study, the *P. pingi* was taken as the research object. Through multi-omics integration combined with histopathology and molecular biology, the potential mechanisms underlying the obstruction of ocular melanin synthesis were systematically analyzed. This not only provides basic targets for molecular marker-assisted breeding of endangered *P. pingi* but also offers new theoretical insights into pigmentation disorders in vertebrates.

## 2. Materials and Methods

### 2.1. Experimental Fish and Sample Collection

Eight healthy one-year-old albino *P. pingi* (group W, 7.50 ± 0.76 cm, 6.28 ± 1.62 g) and eight wild-type *P. pingi* (group C, 8.34 ± 0.45 cm, 8.24 ± 1.64 g) were selected for the experiment. All fish were obtained from an aquaculture base in Mabian County, Leshan City, Sichuan Province, China, where the population had been co-reared under standardized water quality and light conditions for a long term. The experimental fish were acclimated in a recirculating water system for 2 weeks, with parameters maintained at a water temperature of 22 ± 1 °C, pH 7.2 ± 0.3, and dissolved oxygen > 6 mg·L^−1^.

After anesthesia with buffered MS222 (250 mg·L^−1^, Aladdin, Shanghai, China), bilateral eyeballs were rapidly collected under sterile conditions. Samples were fixed in 4% paraformaldehyde for histopathological analysis and stored at −80 °C for molecular biological assays.

### 2.2. Histological Analysis

#### 2.2.1. H&E Staining

In accordance with relevant methods [11], two groups of eye samples fixed in 4% paraformaldehyde were paraffin-embedded. The paraffin-embedded eye samples were then sectioned along the sagittal axis using a Leica RM2235 microtome (Wetzlar, Hesse, Germany) to prepare tissue sections with a thickness of 5 μm. Subsequent to sectioning, the tissue sections were subjected to H&E staining, followed by sealing with neutral resin. Finally, the sealed tissue sections were observed and photographed under a Nikon Eclipse E200 (Tokyo, Japan) with 4× and 40× objectives to examine the pathological changes. Images were captured using a Nikon DS-Fi3 digital camera (Nikon Corporation, Tokyo, Japan).

#### 2.2.2. Oil Red O Staining

After enucleation, the eye samples were immediately rinsed with pre-cooled phosphate-buffered saline (PBS), then embedded in optimal cutting temperature (OCT) compound (Sakura Finetek, Torrance, CA, USA) and snap-frozen in liquid nitrogen. Frozen blocks were sectioned along the sagittal axis using a Leica CM1950 cryostat (Leica Biosystems, Wetzlar, Hesse, Germany) at a thickness of 8 μm. After thawing the frozen sections, the Oil Red O staining protocol was performed as described previously [12] (sections were fixed, washed, and dried first, then stained in the dark for 8 min, followed by differentiation, counterstaining, bluing, and sealing with glycerol gelatin). Following the procedure outlined in [13], the content of carotenoids was then examined and analyzed. In brief, Oil Red O is a lipid-soluble dye; areas rich in carotenoids stain bright red, whereas non-carotenoid tissues appear blue-purple after hematoxylin re-staining. The location of the red signal reflects the distribution of carotenoids, and its intensity is related to the content.

#### 2.2.3. Masson–Fontana Staining

Following the procedure outlined in [14], the Masson–Fontana staining was performed. Key steps were as follows: Working solution (Masson–Fontana Solutions A and B, adjusted to slight turbidity, filtered) was used to incubate sections at 4 °C in the dark; sections were then washed, developed with Solution C for 10 min, and counterstained with fresh Van Gieson (VG) solution (0.5% acid fuchsin:1% picric acid = 1:9). After dehydration, xylene clearing, and glycerol gelatin sealing, samples were examined via a Nikon Eclipse E200 microscope (Tokyo, Japan) (different colors correspond to specific cell/tissue types: black for melanin/argyrophilic cells, red for collagen, and yellow for muscle fibers/erythrocytes).

### 2.3. Total RNA Extraction

Total RNA was isolated from eye samples (3 samples per group) using Trizol reagent (Takara Bio, Ohtsu, Japan) following the manufacturer’s instructions. The concentration and purity of the RNA were detected by Nanodrop2000 (Thermo, Waltham, MA, USA), while agarose gel electrophoresis was applied to assess RNA integrity, and Agilent 2100 (Santa Clara, CA, USA) was utilized to determine the RNA Integrity Number (RIN) value. Samples meeting the standards (total RNA ≥ 1 μg, RNA concentration ≥ 45 ng μL^−1^, 1.8 ≤ OD260/280 ≤ 2.2, 2.0 ≤ OD260/230 ≤ 2.2) were used for subsequent tests. After total RNA extraction, eukaryotic mRNA was enriched by oligo (dT) beads. These beads specifically bind to the poly(A) tail of eukaryotic mRNA, separating it from other RNAs (such as rRNA or tRNA) to focus downstream experiments on mRNA.

### 2.4. Transcriptome Analysis

#### 2.4.1. Library Preparation and Quality Inspection

RNA-seq was performed on the Illumina NovaSeq X Plus sequencing platform by Gene Denovo Biotechnology Co., Ltd. (Guangzhou, China). To ensure data quality and the accuracy of subsequent analysis results, the FastQC software (v0.12.1) [15] was employed to conduct quality control on the raw sequencing data. The RNA-seq data generated in this study are publicly accessible in the Genome Sequence Archive (GSA) in the National Genomics Data Center, China National Center for Bioinformation/Beijing Institute of Genomics under the accession number CRA028487.

#### 2.4.2. Assembly and Annotation

After quality control, clean reads (filtered from raw data to remove adapters, low-quality reads, and ambiguous bases) were used for de novo assembly with Trinity software (v2.15.2) [16]. This process involves constructing de Bruijn graphs from short reads, assembling contiguous sequences (contigs), clustering contigs into transcript isoforms, and collapsing redundant transcripts to generate non-redundant Unigene sequences representing unique genes.

Then Unigene sequences were first aligned against the non-redundant (Nr) protein sequence database using NCBI BLAST (V2.14.0) (https://blast.ncbi.nlm.nih.gov/Blast.cgi) (accessed on 1 March 2025) with an e-value threshold of <0.00001 to identify homologous proteins. The functional annotations of these homologous proteins were then mapped to the Gene Ontology (GO, https://www.geneontology.org/) (accessed on 11 March 2025) and Kyoto Encyclopedia of Genes and Genomes (KEGG, https://www.genome.jp/kegg/) (accessed on 11 March 2025) databases to obtain GO terms and KEGG pathway information corresponding to the Unigenes, thereby acquiring their protein functional annotation details.

#### 2.4.3. Expression Level Statistics and Sample Relationship Analysis

The assembled Unigene was quantified using RSEM software (v1.3.2) [17]. Expression levels were presented as raw read counts and Fragments Per Kilobase of transcript per Million mapped fragments (FPKM) values. Based on the expression data, principal component analysis (PCA) was performed using R (http://www.r-project.org/) (accessed on 17 March 2025).

#### 2.4.4. Analysis of Differentially Expressed Genes (DEGs) and Functional Enrichment

RNA differential expression analysis was performed using DESeq2 software (v3.11) [18]. The screening criteria for significant DEGs were as follows: |log_2_FC| > 1 and FDR < 0.05. Hierarchical clustering of the expression patterns of DEGs was conducted, and after z-score normalization, the clustering results were visualized using a heatmap. Transcripts of DEGs were mapped to the GO database (http://www.geneontology.org/) (accessed on 24 March 2025) and KEGG database (https://www.genome.jp/kegg/) (accessed on 26 March 2025) to identify GO terms and pathways that were significantly enriched in DEGs compared to the whole-genome background. The hypergeometric test was used to evaluate the enrichment significance of DEGs in GO terms and pathways, and the Benjamini–Hochberg method was applied for multiple testing correction.

### 2.5. Proteome Analysis

#### 2.5.1. Sample Preparation and Data-Independent Acquisition (DIA) Data Collection

The protein analysis was performed on the Orbitrap Exploris 480 high-resolution mass spectrometer (Thermo, Waltham, MA, USA) by Gene Denovo Biotechnology Co., Ltd. Tissue samples were pretreated using the iST sample preparation kit (PreOmics, Planegg, Germany), involving protein extraction, denaturation, reduction and alkylation, enzymatic digestion, and desalting of peptides. Mass spectrometry data were acquired in DIA mode using this system coupled with a tandem EASY-nLC 1200 system.

#### 2.5.2. Qualitative and Quantitative Analysis and Annotation of Proteins

The raw mass spectrometry data were quality-controlled using QuiC software (v2.0) [19,20], with a Benjamini–Hochberg false discovery rate (FDR) cutoff of 1% applied at both the precursor and protein levels. Protein quantification normalization was performed by using the Pulsar software (v4.0.0) [21] and the local normalization method. The average top 3 filtered peptides which passed the 1% q-value cutoff were used to calculate the major group quantities. The protein functions and classification were analyzed based on searches against the GO database and KEGG database.

#### 2.5.3. Sample Relationship Analysis and Difference Analysis of Differentially Expressed Proteins (DEPs)

PCA was performed with R package (V4.4.1) models (http://www.r-project.org/) (accessed on 2 April 2025) to analyze inter-sample relationships. The parameters of DIA mass spectrometry analysis were as follows: (1) MS: scan range (*m/z*) = 350–1200; resolution = 120,000; AGC target = 1 × 10^6^; maximum injection time = 50 ms; (2) HCD-MS/MS: resolution = 30,000; AGC target = 1 × 10^6^; collision energy = 32; stepped CE = 5%. (3) DIA was performed with a variable isolation window, and each window overlapped 1 *m/z*, and the window number was 60. DEPs were analyzed with FDR correction, followed by stringent filtering (|log_2_FC| > 1 and FDR < 0.05).

#### 2.5.4. Go and KEGG Enrichment Analysis

Transcripts of DEPs were mapped to the GO database and KEGG database to identify GO terms and pathways that were significantly enriched in DEPs compared to the whole-genome background. The calculated *p*-value was adjusted using FDR correction, taking FDR ≤ 0.05 as the threshold. GO terms and pathways meeting this condition were considered significantly enriched among the DEPs.

### 2.6. Gene Set Enrichment Analysis (GSEA)

GSEA was performed using GSEA software (v4.2.3) to identify significantly enriched pathways from the GO and KEGG databases. This analysis utilized two distinct input matrices: (1) a gene expression matrix derived from RNA-seq data, and (2) a protein abundance matrix generated by mass spectrometry quantification. Significantly enriched pathways were defined based on the normalized enrichment score (NES) with FDR threshold of <0.05 for both transcriptomic and proteomic data.

### 2.7. Real-Time Quantitative PCR (qRT-PCR) Analysis

To verify the transcriptome data and evaluate the expression of related genes, qRT-PCR analysis was conducted (*n* = 3). Primer design was carried out using Premier (v6.0) [22], and the relevant primer sequence is listed in Supplementary Table S1. The PCR reaction system and thermal cycling conditions were set based on the methodologies established by Qin et al. [23]. Ct values from *P. pingi* gene expression were normalized to Ct levels of e*EF1-α* [24,25], and the relative expression of genes was estimated by the 2^^(−ΔΔCT)^ method. The assays were performed on a real-time fluorescence quantitative PCR system (Bio-Rad, Hercules, CA, USA).

### 2.8. Data Analysis and Statistics

In this study, all data were presented as mean ± SD (standard deviation). Statistical difference was analyzed by SPSS 27.0 software (IBM Corp., Chicago, IL, USA). Charts were drawn by GraphPad Prism (v8.0, San Diego, CA, USA) and Adobe lllustrator (v 28.7.8, San Jose, CA, USA). The experimental data were first subjected to normality tests. Then, independent sample t-tests were used to evaluate the significance of the differences, and the significance level was set as *p* < 0.05.

## 3. Results

### 3.1. Clinical and Histological Characteristics

**Figure 1:**
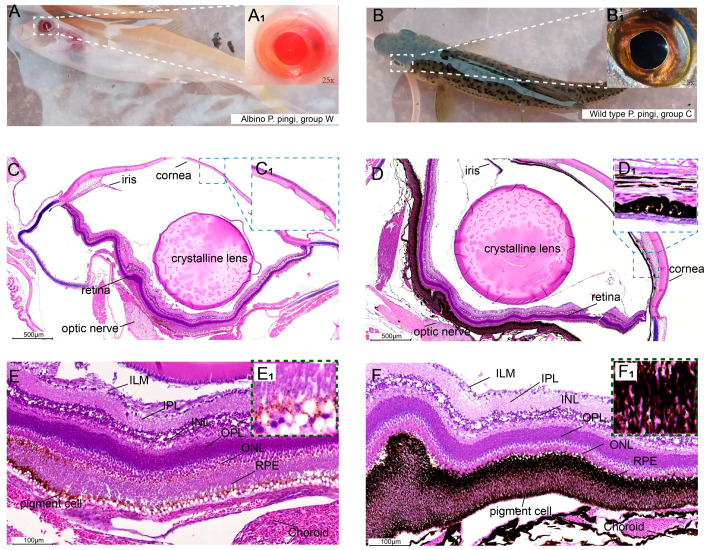
Clinical and H&E staining results. (**A**) Clinical phenotype of group W. (**B**) Clinical phenotype of group C. (**C**) H&E staining of the eyeballs in group W, with a lack of pigment granules. **C_1_**: Magnification of the blue box in Figure C. (**D**) H&E staining of the eyeballs in group C, with rich pigment granules. **D_1_**: Magnification of the blue box in Figure D. (**E**) Retina of group W; **E_1_**: A small number of brown-yellow granules. (**F**) Retina of the group C; **F_1_**: A large number of melanin granules. ILM: internal limiting membranes. IPL: inner plexiform layer. INL: internal nuclear layer. OPL: outer plexiform layer. ONL: outer nuclear layer. RPE: retinal pigment epithelial layer.

The albino *P. pingi* (group W) showed entirely white skin without spots, and the eyeballs and pupils were red (Figure 1A). In contrast, the wild type (group C) exhibited a dark brown body surface with black spots and black eyeballs (Figure 1B). Histological analysis revealed significant differences in eye tissue structures between group W and group C. Specifically, the pigment granule density in the cornea and retina of group W was significantly lower than that in group C (Figure 1C), with the cornea almost completely lacking pigment granules (Figure 1(C_1_)). Only a small number of brown-yellow pigment cells were observed in the RPE layer of group W (Figure 1(E,E_1_)). By contrast, the wild type showed abundant black pigment granules in both the cornea and retina (Figure 1D,F).

### 3.2. Multi-Omics Difference Analysis

**Figure 2:**
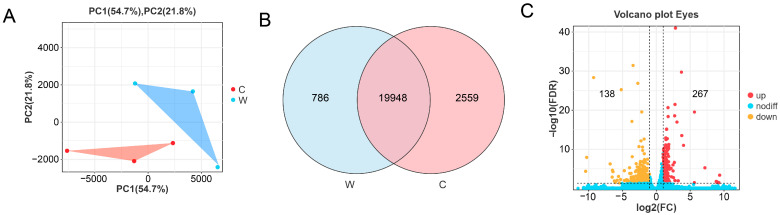
Relationship analysis and basic differences analysis. (**A**) PCA of two groups. (**B**) The Venn diagram shows common genes and specific genes between two groups. (**C**) The volcano plot shows the distribution of DEGs between two groups. (Red dots indicate up-regulated DEGs, orange dots indicate down-regulated DEGs, and blue dots indicate genes with no difference.)

PCA results showed that albino samples and wild-type samples clustered into independent groups (Figure 2A). Venn analysis (Figure 2B) revealed a substantial number of shared and unique genes between the two groups. DEGs between the two groups included 138 downregulated genes and 267 upregulated genes (Figure 2C).

**Figure 3:**
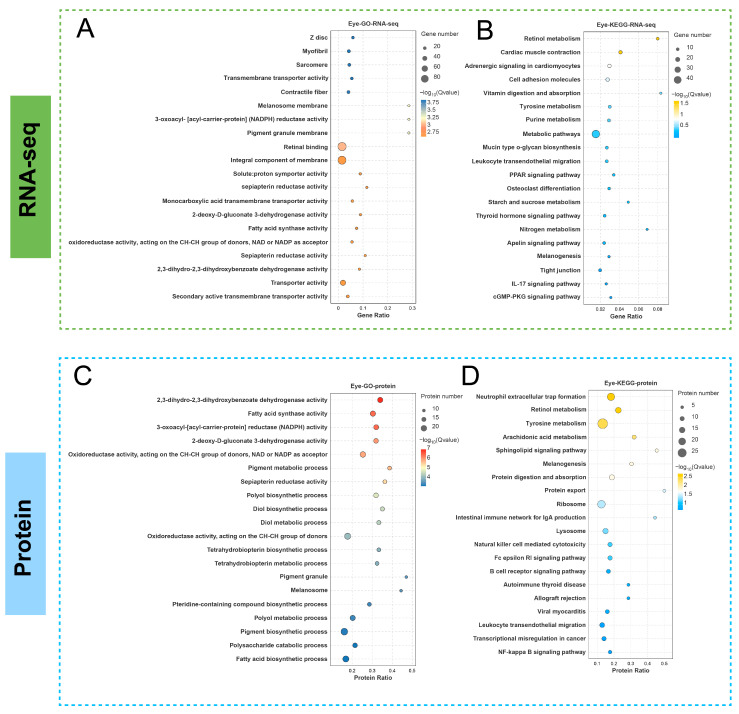
GO enrichment and KEGG enrichment analysis of DEGs in the transcriptome and proteome. (**A**,**B**) GO enrichment and KEGG enrichment analysis of DEGs between two groups in transcriptome, respectively. (**C**,**D**) GO enrichment and KEGG enrichment analysis of DEPs between two groups in proteome, respectively. The vertical axis represents the name of the pathway, and the horizontal axis (Rich factor) represents the ratio of sample number to background number. The size and color of the dots represent the number of genes and the adjusted *p*-value for each pathway, respectively. The smaller the *p*-value (the darker the color), the stronger the association between the pathway and the studied phenotype, and the higher the credibility.

Transcriptomic analysis showed that DEGs between the two groups were primarily enriched in GO terms related to actin structures (Z disk, myofibril, sarcomere), transmembrane transporter activity, and pigment granules (melanosome membrane) (Figure 3A). In the KEGG database, these DEGs were predominantly enriched in retinol metabolism and tyrosine metabolism pathways (Figure 3B). Specifically, within the tyrosine metabolism pathway (Figure S1), key DEGs included those encoding rate-limiting enzymes such as TYR, tyrosinase-related protein 1 (TYRP1), and microphthalmia-associated transcription factor (MITF).

Proteomic analysis yielded that DEPs were enriched in GO terms associated with NADPH activity and pigment metabolic processes, including 3-oxoacyl-[acyl-carrier-protein] reductase (NADPH) activity, pigment metabolic process, and melanosome (Figure 3C). KEGG analysis showed enrichment in retinol metabolism, tyrosine metabolism, and melanogenesis pathways (Figure 3D). The specific DEPs included LRAT, ADH, Rpe65, BCO1, DCT, TYR, and TYRP1 (Figures S2–S4).

Given the significant differences in both DEGs and DEPs related to melanin synthesis, retinol metabolism, and cytoskeletal transport processes between the two groups, we specifically screened the corresponding related molecules and pathways for subsequent analysis.

### 3.3. Analysis of Pathways Related to Melanin Synthesis

**Figure 4:**
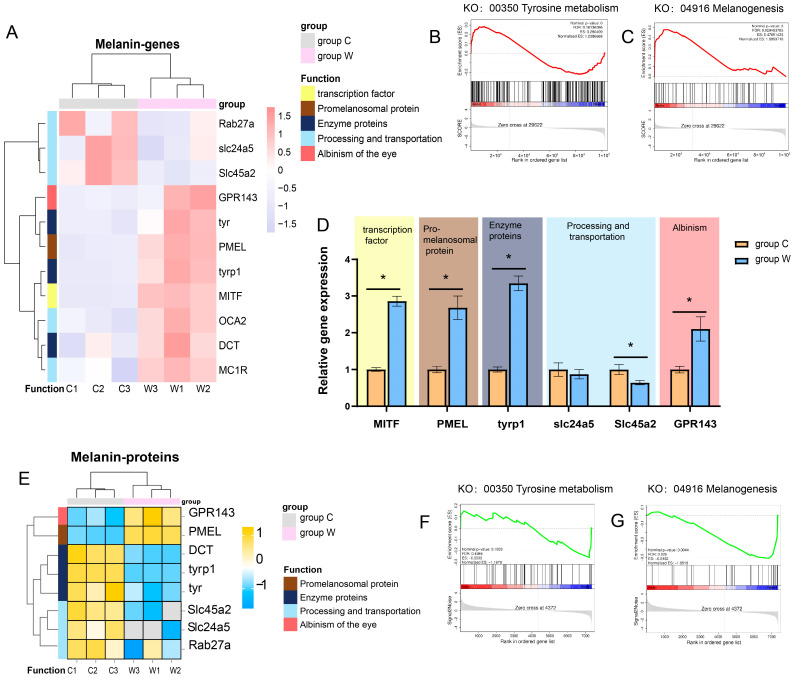
Transcriptome and proteomic results related to melanin synthesis. (**A**) Heatmap of melanin synthesis-related genes with z-score normalized expression levels between the two groups. (**B**,**C**) GSEA of tyrosine metabolism (KO: 00350) and melanogenesis (KO: 04916) in the transcriptome, respectively. (**D**) Expression levels of key genes regulating melanin synthesis. (**E**) Heatmap of melanin synthesis-related proteins with z-score normalized abundance between the two groups. (**F**,**G**) GSEA of tyrosine metabolism (KO: 00350) and melanogenesis (KO: 04916) in the proteome, respectively. * *p* < 0.05, *n* = 3.

Transcriptomic analysis showed that most melanin synthesis-related gene transcripts were upregulated in group W compared to the control group (Figure 4A), including *MITF*, *DCT*, *OCA2*, *TYR*, and *TYRP1*. GSEA revealed significant activation of tyrosine metabolism (KO: 00350) (Figure 4B) and melanogenesis (KO: 04916) (Figure 4C) in group W. qRT-PCR validation (Figure 4D) showed that *MITF, PEML, TYRP1*, and *GPR143* were significantly upregulated in group W, whereas the solute carrier gene SLC45a2 was significantly downregulated.

Interestingly, proteomic results showed upregulation of GPR143 and PEML in group W, but significant downregulation of protease modification and transport-related proteins (SLC45A2, SLC24A5, TYR, TYRP1) (Figure 4E). Moreover, GSEA indicated significant inhibition of tyrosine metabolism (KO: 00350) (Figure 4F) and melanogenesis (KO: 04916) (Figure 4G) pathways at the protein level in group W compared to the control.

### 3.4. Overactivation of Retinol Metabolism in Albino Individuals

**Figure 5:**
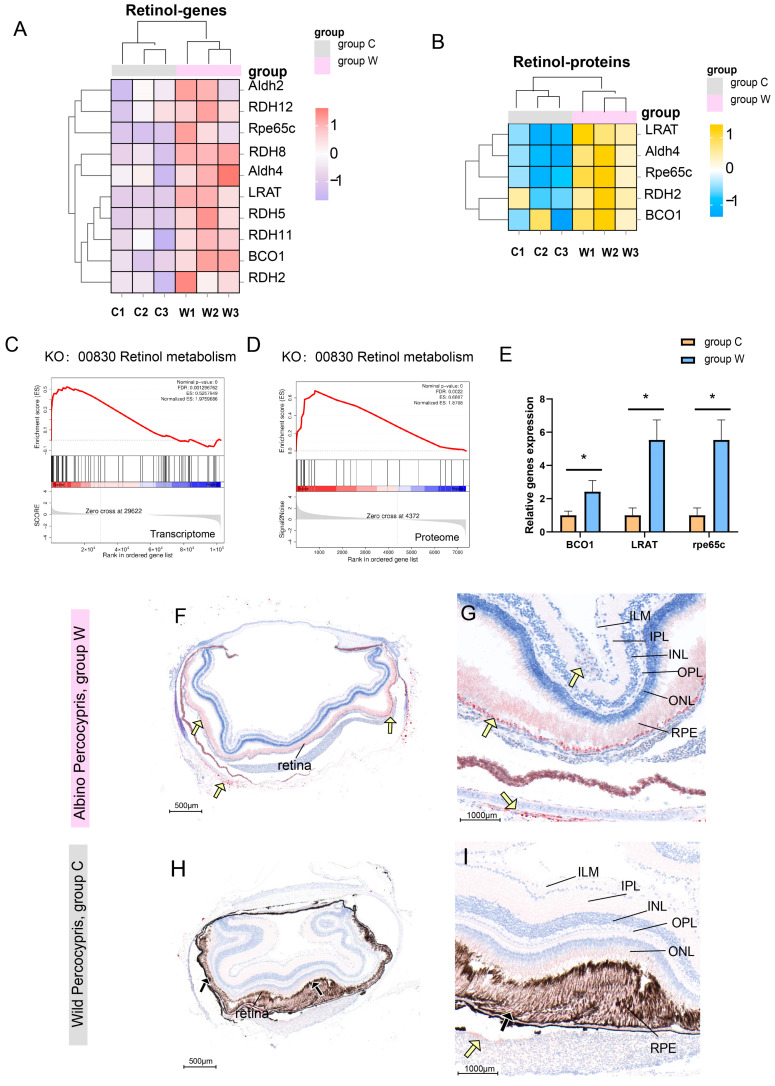
Results related to excessive activation of retinol metabolism. (**A**) Heatmap of retinol metabolism-related genes with z-score normalized expression levels between the two groups. (**B**) Heatmap of retinol metabolism-related proteins with z-score normalized abundance between the two groups. (**C**) GSEA of retinol metabolism (KO: 00830) in the transcriptome. (**D**) GSEA of retinol metabolism (KO: 00830) in the proteome. (**E**) Expression levels of key genes regulating retinol metabolism. (**F**–**I**) Oil Red O staining of eye tissues. (**F**) Eye tissues of group W lacked melanin granules but contained abundant carotenoid particles (yellow arrows). (**G**) Abundant carotenoid particles (yellow arrows) were distributed in the RPE of group W, with occasional carotenoid particles in the ILM. (**H**) Control group eye tissues showed abundant melanin (black arrows). (**I**) In the control group, occasional carotenoid particles were observed (yellow arrows). * *p* < 0.05, *n* = 3. ILM: internal limiting membranes. IPL: inner plexiform layer. INL: internal nuclear layer. OPL: outer plexiform layer. ONL: outer nuclear layer. RPE: retinal pigment epithelial layer.

Transcriptomic and proteomic results consistently showed significant upregulation of key retinol metabolism-related molecules (such as RDH12, LRAT, RPE65C, and BCO1) at both transcript and protein levels in group W compared to the control group (Figure 5A,B). GSEA further confirmed significant activation of the retinol metabolism pathway (KO: 00830) at both omics levels (Figure 5C,D). qRT-PCR validation demonstrated significant upregulation of *BCO1, LRAT,* and *Rpe65c* in group W (Figure 5E). Oil Red O staining revealed abundant melanin and occasional carotenoid particles in the control eye tissues (Figure 5H,I). In contrast, albino eye tissues lacked melanin granules, but the number of carotenoid granules was higher than that of the wild type. These granules were distributed in the RPE and ILM (Figure 5F,G).

### 3.5. Impaired Cytoskeletal Transport Function in the Albino Individuals

**Figure 6:**
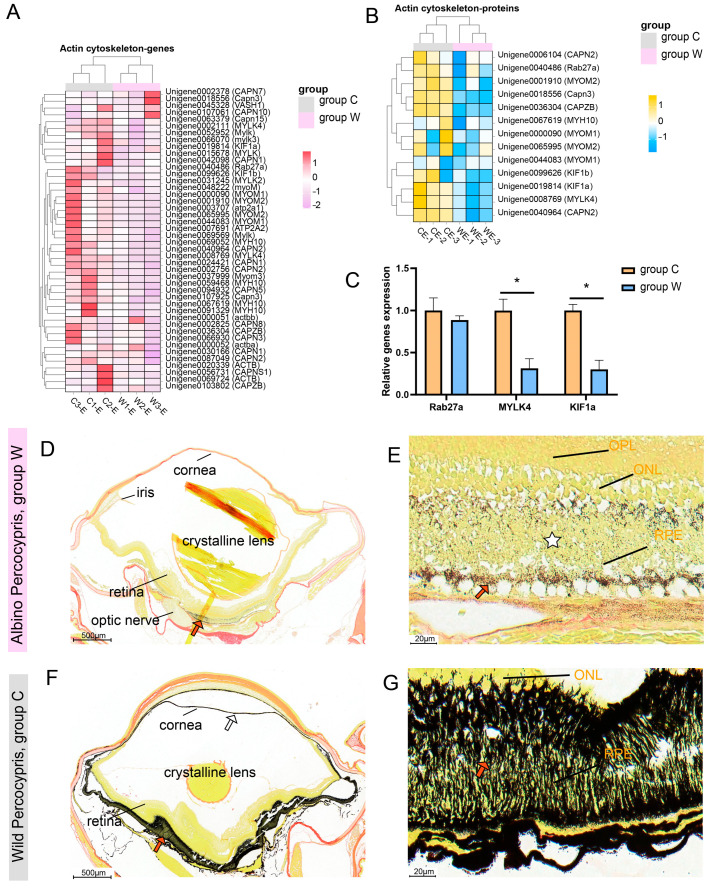
Results related to abnormal cytoskeletal function. (**A**) Heatmap of cytoskeletal-related genes with z-score normalized expression levels between the two groups. (**B**) Heatmap of cytoskeletal-related proteins with z-score normalized abundance between the two groups. (**C**) Expression levels of key genes regulating the cytoskeleton. (**D**–**G**) Masson–Fontana staining of eye tissues. (**D**) Sparse melanin granules (arrows) in eye tissues of group W. (**E**) Melanin in the RPE of group W predominantly deposited in the basal region (arrows) with reduced apical distribution (star). (**F**) Abundant melanin (arrows) in control group eye tissues. (**G**) Uniform distribution of melanin granules in both basal and apical regions of the control group RPE (arrows). * *p* < 0.05, *n* = 3. OPL: outer plexiform layer. ONL: outer nuclear layer. RPE: retinal pigment epithelial layer.

Transcriptomic and proteomic analyses consistently revealed significant downregulation of actin and microtubule motor-related molecules (CAPZB, MYH10, MYOM2, MYLK4, KIF1a, KIF1b) at both transcript and protein levels in group W compared to the control group (Figure 6A,B). The qRT-PCR verification confirmed that, compared with the wild-type *P. pingi*, the expression levels of *MYLK4* and *KIF1a* in the eye tissues of albino *P. pingi* were significantly decreased, while the expression level of *Rab27a* showed a non-significant downward trend (Figure 6C). Masson–Fontana staining demonstrated that melanin in the RPE of group W was abnormally clustered at the basal region with sparse distribution at the apical region (Figure 6D,E), in contrast to the uniform distribution observed in the control group (Figure 6F,G). These results suggest defective melanin transport.

## 4. Discussion

### 4.1. Abnormal H^+^ Transport Mediated by SLC45A2

SLC45A2 encodes a presumed transporter protein primarily expressed in pigment cells. Mutations in SLC45A2 are associated with oculocutaneous albinism type 4 (OCA4) [26]. Patients with OCA4 exhibit extremely low pigmentation levels, indicating a critical role of SLC45A2 in melanogenesis [27]. One of the key processes in melanogenesis is the neutralization of the acidic environment of the early melanosomes [28]. Tyrosinase (TYR), a key enzyme in melanogenesis, catalyzes the rate-limiting step of melanin synthesis [29], but its activity is minimal at pH < 6 [30]. Therefore, melanogenesis is highly dependent on the pH homeostasis of melanosomes. Studies have shown that SLC45A2 is involved in regulating melanosome pH [26], and SLC45A2 knockout leads to increased acidification of early melanosomes, thereby affecting tyrosinase activity and ultimately limiting melanin synthesis [31].

Through integrative transcriptomic and proteomic analyses, this study identified a characteristic expression pattern of ocular melanin synthesis-related genes in albino *P. pingi*: compared with wild-type controls, core melanogenesis regulatory genes (such as *MITF*, *DCT*, *OCA2*, *TYR*, and *TYRP1*) were significantly upregulated at the transcript level, while the solute carrier gene *SLC45A2* was significantly downregulated. At the protein level, consistent with the transcriptomic trend of *SLC45A2*, SLC45A2 protein was significantly downregulated; notably, TYR and TYRP1 proteins (whose encoding genes were transcriptionally upregulated) also showed significant downregulation.

This “transcription-upregulation but protein-downregulation” decoupling is similar to the regulatory patterns reported in previous studies: Mo et al. [32] conducted a study on the regulation of melanogenesis in mice through IFNG, which showed that TYR can exhibit a “protein upregulation but mRNA unchanged” decoupling due to post-translational modifications (IFNG promotes TYR mature glycosylation and reduces its degradation). Rajan Logesh et al. [33] pointed out that the core pathology of OCA1-4 albinism lies in the degradation of melanogenesis-related proteins (such as MITF, TYR, and ACTH) by post-translational modification defects, ultimately leading to reduced pigmentation. Hu et al. [34] also confirmed that USP13 (a deubiquitinating enzyme ) upregulates MITF-M protein levels by inhibiting its degradation (without altering MITF-M mRNA expression), thereby regulating downstream TYR expression and melanin synthesis. Combined with these prior findings, we hypothesize that the TYR/TYRP1 decoupling observed in albino *P. pingi* indicates that the post-translational modification regulation of pigment cells in the eyes of albino individuals may be abnormal. Our further analysis revealed that although the transcriptional regulatory network of *MITF* and *PEML* was not significantly affected in albino *P. pingi*, the significant downregulation of SLC45A2 (at both transcript and protein levels) raises a critical inference: SLC45A2 inhibition may lead to acidification of the intracellular microenvironment (especially melanosomes) in ocular pigment cells.

However, it is important to clarify that this study did not directly measure the pH of ocular pigment cells in albino *P. pingi*. The link between SLC45A2 downregulation and intracellular acidification is derived from two lines of evidence: First, the well-documented role of SLC45A2 in melanosome pH regulation [26]. Second, the significant downregulation of SLC45A2 is observed in this study. If such acidification occurs, it would likely induce conformational denaturation of TYR [31], thereby blocking the key catalytic step of melanin synthesis. This hypothetical regulatory pathway is highly consistent with the phenotype of the SLC45A2-deficient (underwhite) mouse model, where SLC45A2 loss leads to disrupted TYR processing and impaired intracellular trafficking to melanosomes [35].

To fully validate this inference, future studies should supplement direct experimental data on melanosome pH in albino *P. pingi* ocular tissues (such as using targeted pH-detection techniques). Such data would confirm the causal relationship between SLC45A2 downregulation, melanosome acidification, and TYR inactivation, thereby strengthening the mechanistic conclusion of this study.

### 4.2. Excessive Activation of Retinoid Metabolism Inhibits Tyrosinase Activity

Retinoids (such as retinol, retinal, retinoic acid, and all-trans retinoic acid) play a key role in the regulation of UV-induced melanin metabolism [36]. Studies have confirmed that these substances inhibit pigmentation through a dual mechanism: reducing melanosome transfer to keratinocytes and exerting a depigmenting effect by regulating the tyrosine metabolism pathway to improve abnormal pigmentation [37]. Tyrosinase, a key rate-limiting enzyme in melanogenesis, directly reduces melanin synthesis when its activity is decreased. Sato et al. [38] found in a mouse B16 melanoma cell model that retinol significantly downregulated tyrosinase mRNA levels in a dose-dependent manner, thereby inhibiting melanin production. The melanin-inhibiting effect of retinoids is closely associated with nuclear receptor-mediated transcriptional regulation [39,40]. Heterodimers formed by retinoic acid receptors (RARs) and retinoid X receptors (RXRs) are effective regulators of the cell cycle, differentiation, proliferation, and apoptosis [41]. Baldea et al. [42] observed in primary human melanocyte culture experiments that all-transretinoic acid (ATRA) treatment reduced the proliferation index of melanocytes and inhibited dendritic processes.

This study found that compared with wild-type *P. pingi*, the retinoid metabolic pathway in the eye tissues of albino *P. pingi* was significantly activated. The retinoid metabolism-responsive genes (*BCO1*, *LRAT*, and *Rpe65c*) were upregulated in eye tissues, accompanied by downregulation of tyrosinase protein expression. This suggests that the ocular depigmentation in albino *P. pingi* may be caused by overactivated retinol metabolism, which inhibits key enzymes such as tyrosinase, resulting in the obstruction of tyrosine conversion to L-dopa and insufficient precursors for melanin synthesis.

### 4.3. Cytoskeleton Transport Defect

Melanosomes are specialized intracellular organelles for melanin production and storage in melanocytes, distributed across multiple tissues and organs, including the skin, hair, and eyes [43]. After gradual formation and maturation within melanocytes (stages I–IV), melanosomes rely on a molecular motor system for transcellular transport. Long-distance transport relies on kinesin moving along microtubule tracks, while short-range trafficking is mediated by the actin network (predominantly F-actin) [44]. In RPE of eye tissues, microtubule motor transport mediated by kinesin plays a critical role in delivering melanosomes to the actin-rich apical domain of the RPE cells [45].

Through the combined analysis of transcriptome and proteome in this study, it was found that in the eye tissues of albino *P. pingi* (group W), the transcripts and protein levels of core actin cytoskeleton components (MYH10, MYOM2, MYLK4) and microtubule motor proteins (KIF1a, KIF1b) were significantly downregulated. Masson–Fontana staining showed abnormal aggregation of melanosomes near the basement membrane with sparse apical distribution in RPE cells of group W. These findings indicate that cytoskeletal dysfunction in group W may disrupt transport tracks, leading to melanin granule retention and hindering the transport of pigment granules to the apical region of the RPE layer.

### 4.4. Potential Regulatory Molecular Networks

**Figure 7:**
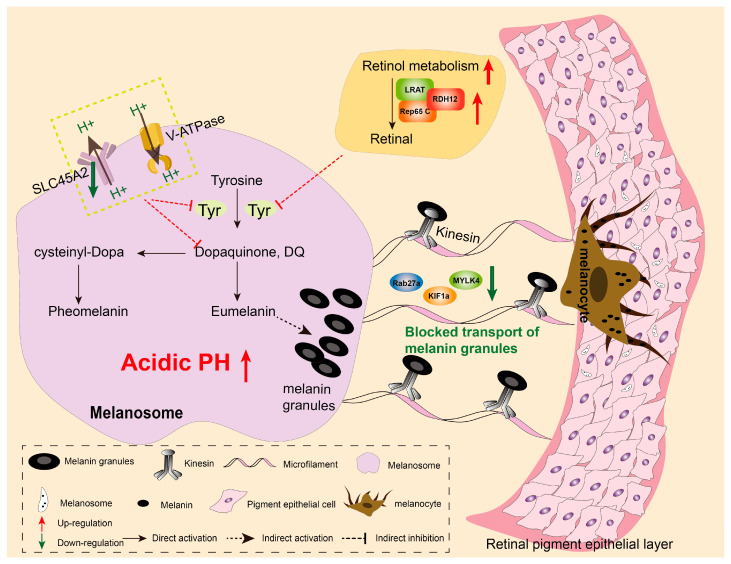
The potential mechanism of impaired melanin synthesis in albino *P. pingi* eyes.

Integrating transcriptomic, proteomic, molecular biological, and histopathological results, this study for the first time constructs a potential mechanism underlying melanin synthesis blockage in albino *P. pingi* eyes (Figure 7). First, downregulation of SLC45A2 in albino eyes reduces H^+^ efflux, creating an intracellular acidic environment that inhibits tyrosinase conformational stability. Second, overactivated retinol metabolism suppresses the activity of key enzymes (such as tyrosinase), leading to insufficient melanin synthesis precursors. Finally, cytoskeletal transport defects cause melanin granules to be retained in melanocyte cell bodies and the basement membrane of the RPE, preventing their transport to the apical region of the RPE and resulting in reduced melanin deposition. Collectively, these three factors lead to the overall failure of the melanin synthesis pathway, ultimately forming the ocular depigmentation phenotype of albino *P. pingi*.

### 4.5. The Limitations of This Study and Future Research Directions

Although this study systematically elucidated the molecular mechanisms of ocular melanin synthesis defects in albino *P. pingi* through integrative multi-omics analysis, several limitations still remain. First, while the study identified a synergistic effect of SLC45A2-mediated H^+^ transport abnormality, overactivated retinol metabolism, and cytoskeletal transport dysfunction leading to melanin deficiency, direct evidence for SLC45A2-regulated melanosome pH remains unvalidated. Additionally, the specific mechanisms by which overactivated retinol metabolism inhibits tyrosinase activity and whether there are specific receptors or signaling pathways have not yet been fully clarified.

Future studies will further explore the post-translational modification mechanisms of SLC45A2 to clarify the regulatory pathways underlying its downregulation in albino individuals. Also, we will utilize small-molecule inhibitors and in vitro models to dissect the specific regulatory pathways of retinol metabolism intervention on tyrosinase activity and the expression of related genes and ultimately evaluate its potential to regulate the eye color of fish.

## 5. Conclusions

Through integrative multi-omics analysis, this study elucidated the potential molecular mechanisms underlying ocular melanin synthesis defects in albino *P. pingi*, specifically involving SLC45A2-mediated H^+^ transport abnormalities, overactivation of the retinol metabolism pathway, and cytoskeletal transport disorder. This research lays a theoretical foundation for in-depth understanding of ocular pigment metabolic disorders in vertebrates.

## Data Availability

The RNA-seq data is available in the Genome Sequence Archive (GSA) in the National Genomics Data Center, China National Center for Bioinformation/Beijing Institute of Genomics via accession number CRA028487.

## Supplementary Materials

The following supporting information can be downloaded at: https://www.mdpi.com/article/10.3390/cells14171377/s1. Table S1. Primer sequences in this study. Figure S1. Tyrosine metabolism (KO: 00350) in the transcriptome. Figure S2. Retinol metabolism (KO: 00830) in the proteome. Figure S3. Tyrosine metabolism (KO: 00350) in the proteome. Figure S4. Melanogenesis (KO: 04916) in the proteome.
