# Supplementary material for: Histopathological Characteristics and Multi-Omics Analysis of Ocular Pigmentation Defects in Albino Percocypris pingi
Source: Cells. 2025 Sep 4;14(17):1377. doi: 10.3390/cells14171377 (PMC12427960; doi:10.3390/cells14171377)
Supplement: Supplementary file 1 [file cells-14-01377-s001.zip › cells-3844858-supplementary.pdf]

Table S1. Primer sequences in this study.

Gene name	primer sequence (5′-3′)	GenBank accession number
MITF	F: CTCGCCGTTGTAGTCTCTCC<br>R: GTTGAGGTCCAGAGTGGTGG	XM_058778880.1
PEML	F: CTGAGAGCCCCATCCACCTA<br>R: AGGCGTGGGTGGGATAGTTT	XM_016290998.1
Tyrp1	F: GGTGCGACGTAATGTCATGC<br>R: CTGGGCTGGACCCATGTAAG	XM_058779617.1
GPR143	F: GACACGCTCCTCCACACAAA<br>R: TGCTGTTTCCAGCGGAGAT	XM_058786580.1
SLC45a2	F: ACTCTGCTATGCCGTTGAGG<br>R: ACGATCGCCCATGTCCTTTT	XM_058758579.1
SLC24a5	F: GCCATTCTGCTGCAAATCCTG<br>R: CGCTGATGAGCTCCAGAGAC	XM_058751641.1
BCO1	F: CCACCTTTGAAAAGCGCTGA<br>R: AATCAGGGTGCTGTCTTCGG	XM_058782192.1
LRAT	F: AGACAGGCGGAAGGAAAACT<br>R: CCAAAGAGTCCACACGGACA	XM_058756332.1
Rpe65c	F: AAACATGGCCAGAGGAGAGC<br>R: GGTTCAAGCCAAACGGTTCC	XM_016291520.1
MYLK4	F: CCAACATCTGGAGAACGGCG<br>R: TCTGCGTCTTTCATCTCGTCTT	XM_058799423.1
KIF1a	F: CAGAGGCAGGTCAACTCCAG<br>R: GCCAGTTCTGTCTCTCGCTT	XM_058749442.1
Rab27a	F:AAACTTCTCCTCAGTCGGTTTG<br>R: ACCGCTGGATTTGTACACCA	XM_058752433.1
eEF1a	F:ACACAGACTTGGACTGTGCC<br>R: GTAGCTTCACTGCTCGGGAC	XM_058762838.1

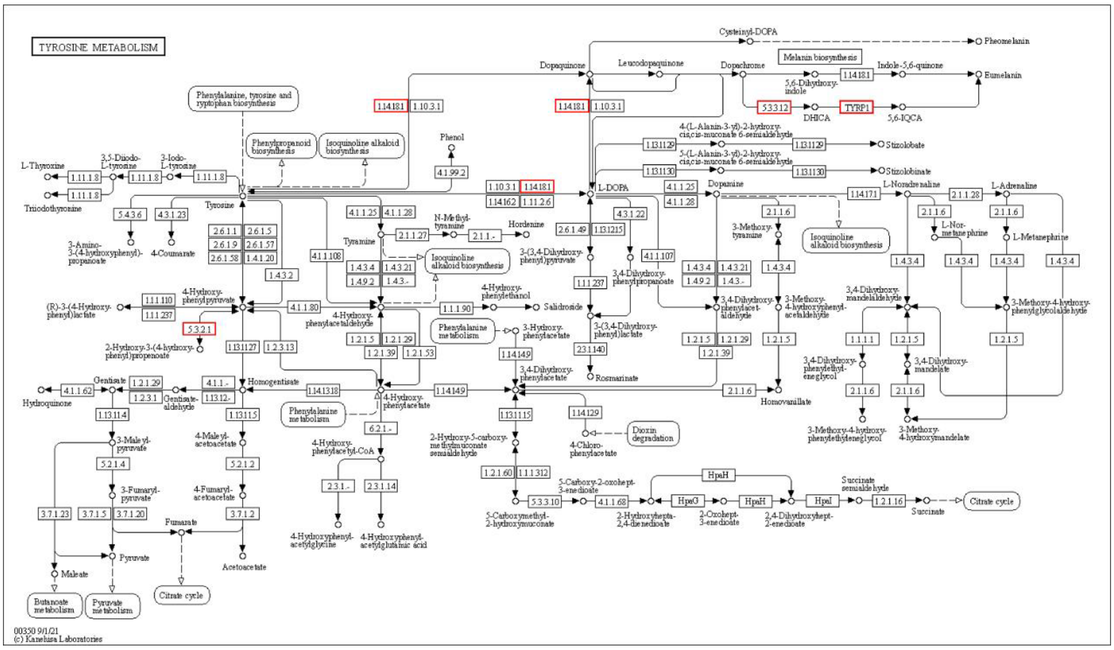

Fig. S1. Tyrosine metabolism (KO: 00350) in the transcriptome

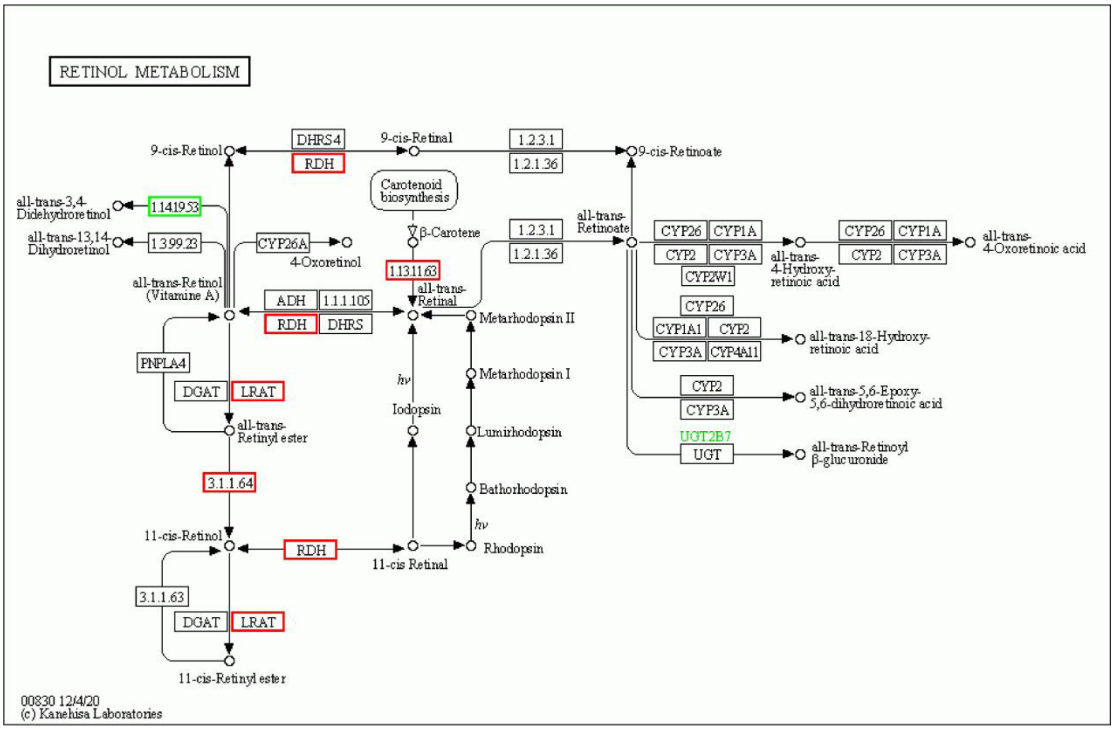

Fig. S2. Retinol metabolism (KO: 00830) in the proteome

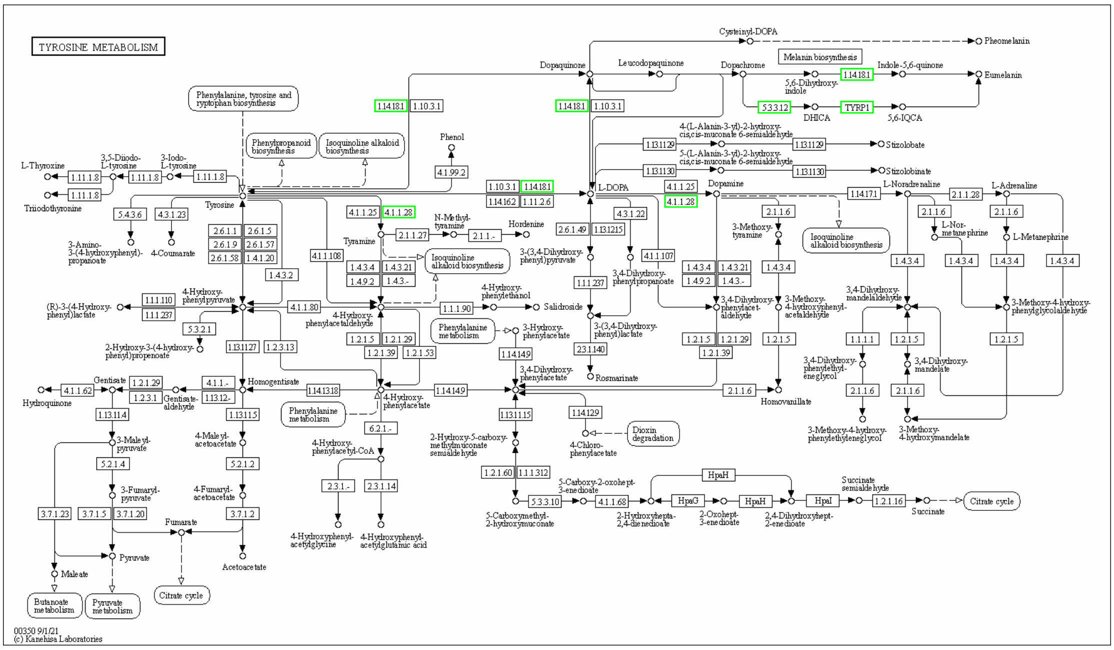

Fig. S3. Tyrosine metabolism (KO: 00350) in the proteome

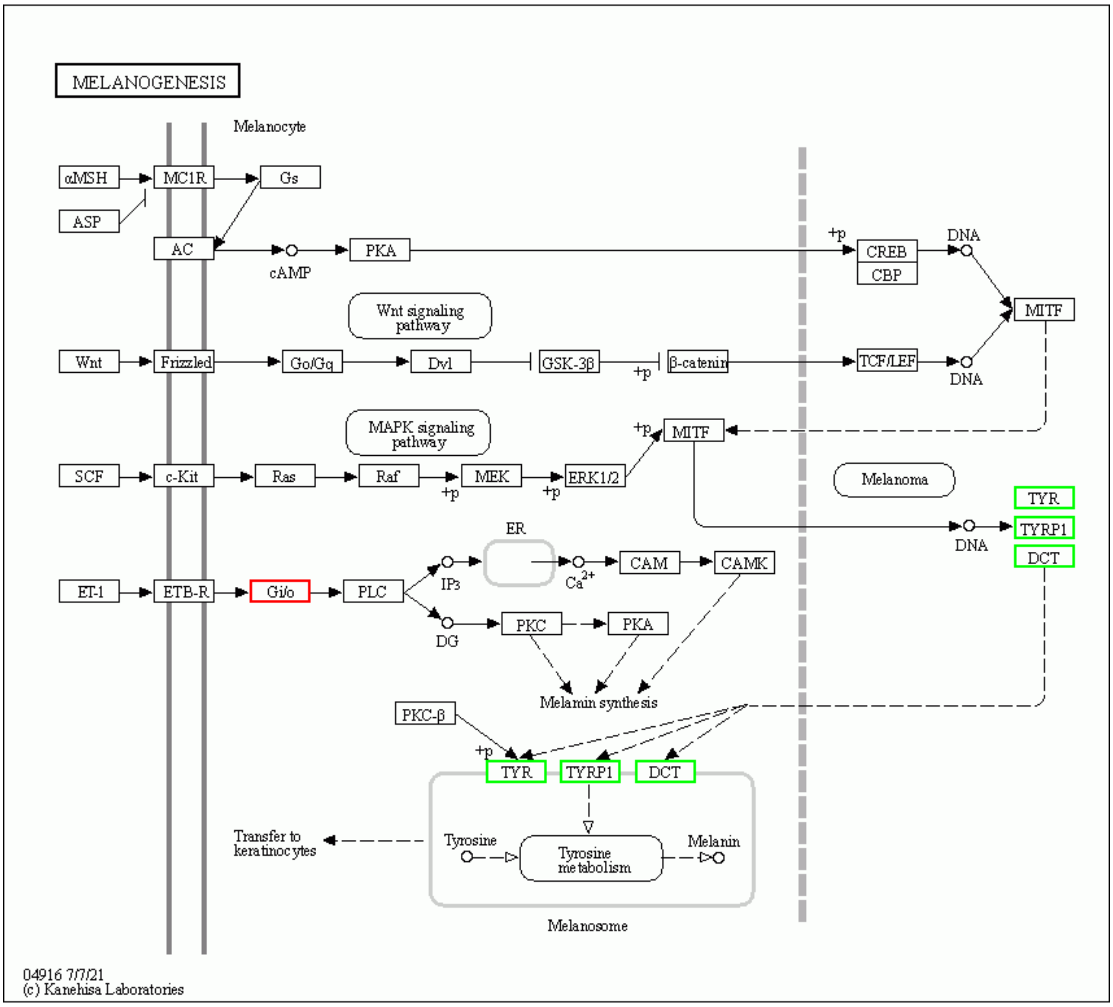

Fig. S4. Melanogenesis (KO: 04916) in the proteome
